# Mid-wall fibrosis on delayed enhancement CMR - a marker for adverse left ventricular chamber remodeling independent of cardiomyopathic etiology

**DOI:** 10.1186/1532-429X-16-S1-O23

**Published:** 2014-01-16

**Authors:** Jiwon Kim, Jonathan D Kochav, Sergey Gurevich, Evelyn M Horn, Parag Goyal, Maya M Petashnick, Anika Afroz, Peter M Okin, Richard B Devereux, Jonathan W Weinsaft

**Affiliations:** 1 Medicine, Weill Cornell Medical College, New York, New York, USA; 2 Medicine, Memorial Sloan Kettering Cancer Center, New York, New York, USA

## Background

Mid-wall fibrosis (MWF) is a hallmark of non-ischemic cardiomyopathy (NICM) that confers increased risk for sudden cardiac death and mortality. The relationship between MWF and left ventricular (LV) remodeling is unknown.

## Methods

The population comprised patients with advanced systolic dysfunction (LVEF ≤ 40%) undergoing CMR (1.5T, General Electric Signa). Ischemic vs. NICM etiology was classified in accordance with established convention based on obstructive CAD on invasive angiography. Delayed enhancement CMR (IR-GRE acquired 10-30 minutes post gadolinium [0.2 mmol/kg]) was used to identify MWF, defined as hyperenhancement confined to the mid-myocardial or epicardial aspect of the interventricular septum. LV mass and chamber volume were quantified by planimetry of contiguous SSFP cine-CMR short axis slices, with ejection fraction (EF) calculated as the proportional difference between end-diastolic (EDV) and end-systolic (ESV) volumes.

## Results

523 patients (61 ± 14 yo, 72% M, 66% ischemic CM) were studied: MWF was present in 16%, and was 6-fold more common in patients with angiographically classified NICM (37% vs. 6%; p < 0.001). Regarding LV remodeling, MWF was associated with higher EDV (134 ± 39 ml vs. 114 ± 34 ml; p < 0.001) and LV mass (102 ± 24 vs. 96 ± 28 gm; p < 0.001) and lower LVEF (26 ± 8% vs. 30 ± 8%; p ≤ 0.05). MWF was nearly 3-fold more common among patients in the highest tertile of EDV vs. the remainder of the population (29% vs. 10%; p < 0.001). Multivariate regression analysis was performed to further assess markers for MWF: Restricted to imaging indices, results (Table 1A) demonstrated EF and EDV to be independently associated with MWF (OR = 1.46, CI 1.03-2.10; p < 0.05, OR = 1.13, CI 1.04-1.20; p < 0.05, respectively) after controlling for mass (OR = 0.95, CI 0.85-1.00; p = 0.29). Regarding clinical variables, results (Table 1B) confirmed a strong association with NICM (OR = 8.4, CI 4.78-14.72; p < 0.001), independent of other clinical indices. A combined model incorporating both clinical and imaging variables demonstrated both NICM and LV volume to be independently associated with MWF even after controlling for EF (Table 1C). Overall strength of the combined clinical/imaging (χ^2 ^= 91.2; p < 0.001) model for MWF was higher than that of isolated clinical (χ^2 ^= 76.7; p < 0.001) and imaging (χ^2 ^= 26.0; p < 0.001) models.

**Table 1:** Multivariate Regression for Prediction of LV MWF

1A. Imaging	(Model χ^2 ^= 26.0, p < 0.001)		
Variable	Odds Ratio	95% Confidence Interval	P
LV Ejection Fraction (per 10% decrement)	1.46	1.03-2.10	< 0.05
LV End-Diastolic Volume (per 10 ml/m2)	1.13	1.04-1.20	< 0.05
Myocardial Mass (per 10 gm/m2)	0.95	0.85-1.00	0.29
			
**1B. Clinical**	(Model χ^2 ^= 76.7, p < 0.001)		

Variable	Odds Ratio	95% Confidence Interval	P
Hypertension	0.10	0.36 - 1.10	0.10
Hypercholesterolemia	0.94	0.59 - 1.78	0.94
Age (years)	0.70	0.99 - 1.00	0.70
Non-Ischemic Etiology	8.4	4.78- 14.72	< 0.001
			
**1C. Integrated Clinical/Imaging**	(Model χ^2 ^= 91.2, p < 0.001)		

Variable	Odds Ratio	95% Confidence Interval	P
LV Ejection Fraction (per 10% decrement)	1.22	0.84- 1.78	0.29
LV End-Diastolic Volume (10 ml/m2)	1.11	1.03 - 1.20	< 0.05
Non-Ischemic Etiology	8.22	4.75 - 14.2	< 0.001

## Conclusions

Among patients with advanced cardiomyopathy, MWF is associated with advanced LV chamber dilation independent of severity of LV dysfunction and cardiomyopathic etiology.

## Funding

National Institutes of Health (K23 HL102249-01).

